# Interstitial deletion of chromosome 1 (1p21.1p12) in an infant with congenital diaphragmatic hernia, hydrops fetalis, and interrupted aortic arch

**DOI:** 10.1002/ccr3.759

**Published:** 2017-01-23

**Authors:** Masitah Ibrahim, Matthew Hunter, Lucy Gugasyan, Yuen Chan, Atul Malhotra, Arvind Sehgal, Kenneth Tan

**Affiliations:** ^1^ Monash NewbornMonash Medical CentreMelbourneVictoriaAustralia; ^2^ Monash GeneticsMonash Medical CentreMelbourneVictoriaAustralia; ^3^ Department of PaediatricsMonash UniversityMelbourneVictoriaAustralia; ^4^ Cytogenetics Laboratory, PathologyMonash Medical CentreMelbourneVictoriaAustralia; ^5^ Anatomical Pathology ServicesMonash Medical CentreMelbourneVictoriaAustralia; ^6^ The Ritchie CentreHudson Institute of Medical ResearchClaytonVictoriaAustralia

**Keywords:** 1p21.1p12, chromosomal deletion, congenital diaphragmatic hernia, etiology, genetics, hydrops fetalis, interrupted aortic arch

## Abstract

We report a case of an infant with congenital diaphragmatic hernia (CDH) and hydrops fetalis who died from hypoxic respiratory failure. Autopsy revealed type B interrupted aortic arch (IAA). Microarray revealed a female karyotype with deletion of chromosome 1p21.1p12. There may be an association between 1p microdeletion, CDH, and IAA.

## Introduction

Congenital diaphragmatic hernia (CDH) is an important cause of neonatal morbidity and mortality. Although generally regarded as a sporadically occurring fetal malformation, it is more common in chromosomal aberrations, and as part of specific genetic syndromes. It is frequently associated with other malformations, particularly involving the cardiovascular system 1. The combination of CDH with a cardiovascular malformation that requires surgical intervention results in dismal prognosis 2. Hydrops fetalis is another rare association with CDH that is known to have near uniform mortality 3. This report describes a case of right‐sided CDH, interrupted aortic arch (IAA), and hydrops fetalis, and the search for potential genetic determinants of non‐isolated CDH.

## Clinical Report

A female infant was born at 37 weeks of gestation via elective cesarean section to healthy, non‐consanguineous Caucasian parents. This was the mother's second pregnancy, conceived via in vitro fertilization (IVF); the first was a miscarriage at 10 weeks of gestation (Fig. 3). Maternal serum screening showed increased risk of aneuploidy (1 in 14), but amniocentesis was declined. An ultrasound scan at 23 weeks of gestation demonstrated right‐sided aortic arch, echogenic focus within the left ventricle, hypoplastic nasal bone, and thickened nuchal fold. Ultrasound scans performed at 28 weeks of gestation revealed right pleural effusion. Two weeks later, a right‐sided CDH with liver herniation and bilateral pleural effusions were identified. Subsequent scans showed significant ascites and subcutaneous edema, consistent with fetal hydrops. Magnetic resonance imaging at 31 weeks of gestation confirmed a moderately severe right‐sided CDH with herniation of 30% of the liver and bilateral pleural effusions. Both kidneys were reported to be abnormally small. The fronto‐occipital hemispheric diameter was on the 10th centile with no intracranial abnormality.

**Figure 1:**
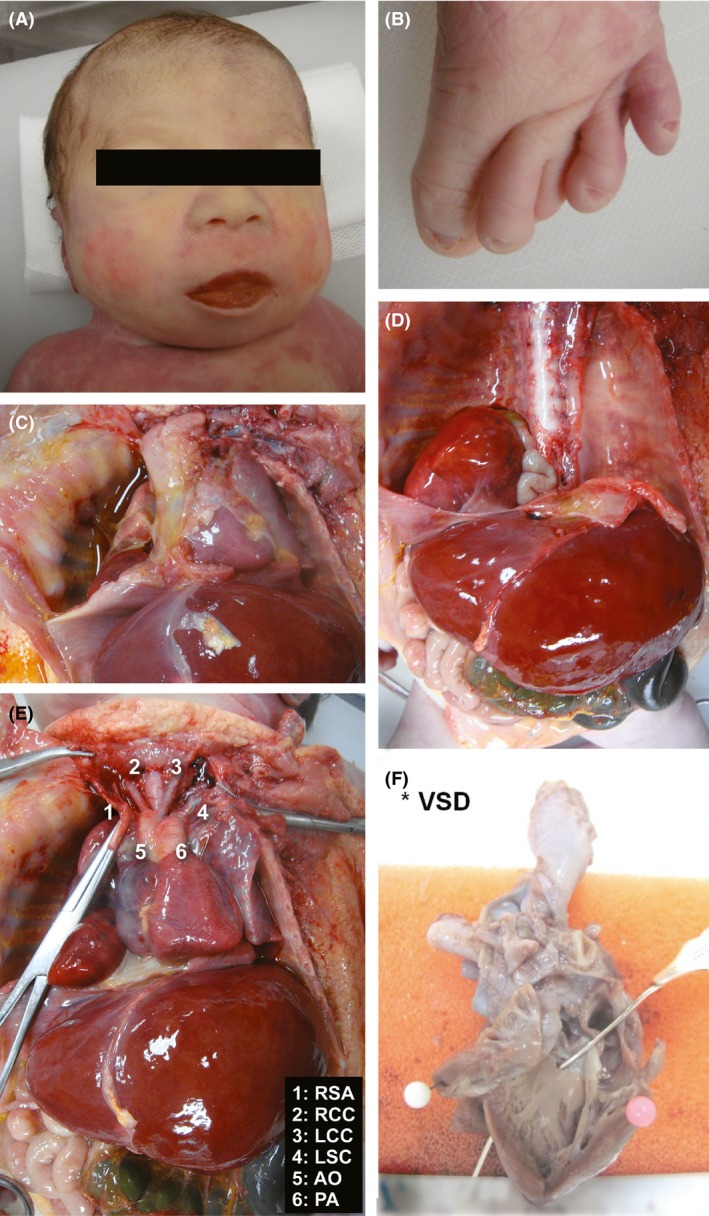
(A) Sloping forehead, depressed nasal bridge, and micrognathia; (B) overlapping of second and fourth toes over the third left toe; (C) hypoplastic right lung; (D) herniated liver and bowel; (E) interrupted aortic arch type B; (RSA, right subclavian artery; RCC, right common carotid; LCC, left common carotid; LSC, left subclavian artery; AO, aorta; PA, pulmonary artery); (F) ventricular septal defect (LV, left ventricle).

The infant was born weighing 3000 g (50th centile), measuring 47.5 cm in length (25th centile), with an occipitofrontal circumference of 31 cm (<10th centile). Facial features included sloping forehead, depressed nasal bridge, and micrognathia (Fig. 1). There was overlapping of the second and fourth toes over the third left toe. The infant was intubated at birth and managed for persistent pulmonary hypertension with intravenous prostaglandin E_2_, inhaled nitric oxide, and inotropes. Chest and peritoneal drains were inserted immediately after birth. The initial hemoglobin was 128 g/L, and infant's blood group was Group A Rh positive. The direct antiglobulin test was negative. Despite maximal intensive care measures, the infant remained in oxygenation failure since birth and developed intractable metabolic acidosis. Care was withdrawn in consultation with the family. The infant survived for 30 h. There was no family history of similar problems.

Postmortem examination confirmed the right‐sided diaphragmatic defect with herniation of right lobe of liver and loops of small bowel into the right pleural cavity, hypoplastic right lung, and pleural effusion (Fig. 1). Histology of the lungs showed patchy bronchopneumonia with formation of hyaline membranes. There was type B interruption of the aortic arch distal to the right and left common carotid arteries; the left subclavian artery arising from the descending aorta, distal to a widely patent ductus arteriosus. The innominate vein was absent and replaced by a persistent left superior vena cava, which drained into the coronary sinus of the right atrium. A perimembranous ventricular septal defect was present, measuring 1–2 mm. Both kidneys were of normal size, cortex and medulla with well‐demarcated corticomedullary junctions (despite the antenatal MRI report). In addition, both ureters and bladder were normal. No abnormalities were found in the other organ systems.

**Figure 2:**
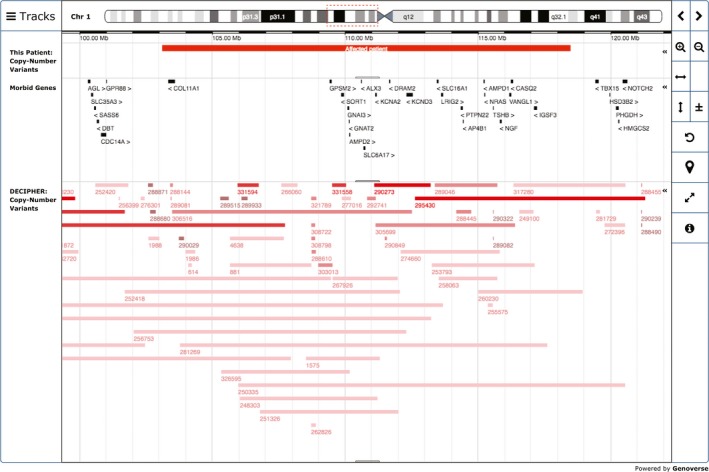
DECIPHER diagram for interval Chr1:103114239‐118470102.

Microarray analysis of DNA extracted from peripheral blood was performed on an Agilent SurePrint Custom G3 CGH+SNP (60K) platform (0.2 Mb resolution) and analyzed using CytoGenomics software (Agilent Technologies Mulgrave VIC, Australia). This revealed a female molecular karyotype with a 15.36 Mb deletion of 1p21.1p12 (deletion coordinates arr[hg19] 1p21.1p12(103,114,239‐118,470,102)x1 dn) involving more than 100 RefSeq genes (Fig. 2). Parental chromosome was normal indicating de novo origin for the deletion.

## Discussion

There is very little published literature about disease association with interstitial deletions of chromosome 1 between 1p12 and 1p21. There have been five case reports of 1p deletions similar to our case 4, 5, 6, 7, 8. Prominent features include short stature, dysmorphic appearance, and neurodevelopmental delay (Table 1). Our patient shared some of these dysmorphic features. Neurodevelopmental assessment was not possible. IAA type B was reported in one case 7. In their 25‐year review of CDH management, Benjamin et al. identified an infant girl with chromosome 1p deletion (46,XX,‐p + der(1)); no further details were reported 9. Our report represents the second detailed report of an association between 1p deletion and CDH.

**Table 1:** Comparison of existing published reports of clinical cases

Features	Dockery 1991	Tabata 1991	Mattia 1992	Stockton 1997	Bisgaard 2007	Our Patient	Totals
Deletion	1p13.2p22.3	1p13.3p22.3	1p13.3p22.3	1p21p22.3	1p13.1p21.1	1p12p21.1	7
Gender	F^a^	F	M	F	F	M	4F/2M
Physical							
Digital anomalies	+	+	−	+	+	+	6/7
Cranial anomalies	+	−	+	+	+	+	6/7
Nasal anomalies	−	+	+	+	+	+	5/7
Ear anomalies	+	−	+	+	+	−	5/7
Foot anomalies	+	+	+	−	+	−	5/7
Short stature	+	+	−	−	+	−	4/7
Neck anomalies	−	+	−	+	+	−	3/7
Micrognathia	−	+	+	−	−	+	3/7
Palate anomalies	−	+	−	+	+	−	3/7
Strabismus	+	−	−	−	+	−	3/7
Vertebral anomalies	−	−	+	+	−	−	2/7
Eyelid anomalies	−	+	+	−	−	−	2/7
Low hairline	−	+	−	−	+	−	2/7
Nipple anomalies	−	+	−	+	−	−	2/7
Reduced lung volume	−	−	−	+	−	+	2/7
Cardiovascular							
IAA type B	−	−	−	+	−	+	2/7
Development							
Developmental delay	+	+	+	NA	+	NA	5/5
Hypotonia	−	+	+	NA	+	NA	3/5
Spasticity	+	−	−	NA	+	NA	3/5
Hearing loss	+	−	−	NA	−	NA	2/5
Seizures	−	+	−	−	+	−	2/7
Other							
	−	TOF, Arh	−	Short nails	Colobomata	CDH	
				Radial hypoplasia	CVI		
				Rib anomalies			
				Small kidneys			

a Monozygotic twins.

NA, not assessed; TOF, tetralogy of Fallot; Arh, Arrhythmias; CVI, central visual impairment; CDH, congenital diaphragmatic hernia.

Conditions well known to be associated with CDH include the following: Pallister–Killian syndrome 10, Cornelia de Lange syndrome 11, Donnai–Barrow syndrome 12, Denys–Drash syndrome 13, Matthew–Wood syndrome 14, Beckwith–Wiedemann syndrome 15, Simpson–Golabi–Behmel syndrome 16, Kabuki syndrome 17, Fryns syndrome 18, and PAGOD syndrome 19, but none of these fit the phenotype of our patient. Special mention needs to go to Fryns syndrome. While our patient would be classified as having “atypical Fryns syndrome” in the classification by Lin et al., we do not think that our patient had sufficient other contributory dysmorphic features to be diagnosed with Fryns syndrome 20. Holder et al. described an extensive list of structural chromosomal anomalies in patients with CDH; however, 1p microdeletion was not identified as a putative site of anomaly apart from the single case reported by Benjamin et al. 9, 21.

A search of the OMIM database (http://genome.ucsc.edu) 22 revealed 20 disease‐causing genes and 84 non‐disease‐causing genes within the 1p band in question (Table S1). Among the disease‐causing genes present within the interval of interest, none matched the potential candidate genes for CDH listed by Brady et al. 23. A search on DECIPHER v8.0 (https://decipher.sanger.ac.uk) 24 revealed 106 patients, 44 of whom had deletions and 62 with duplications of the affected interval in chromosome 1p. Of interest, Decipher ID 281269 had CDH.

**Figure 3:**
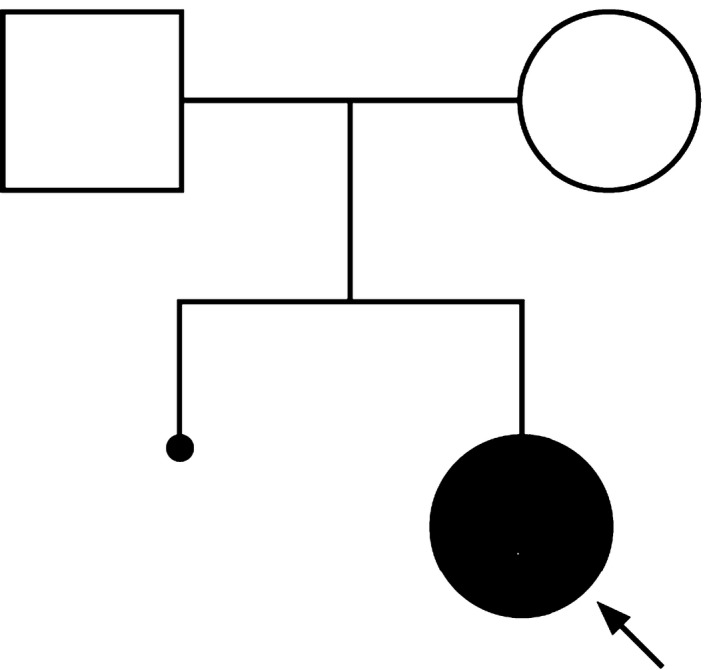
Family pedigree chart.

A review of cardiac defects by the Congenital Diaphragmatic Hernia Study Group showed that the incidence of hemodynamically significant congenital heart disease in patients with CDH is 10.6% 25. Together with ventricular septal defect (VSD) and univentricular anatomy, aortic arch obstruction made up for almost 75% of the cardiac defects observed. Of note, there was a consistent trend toward more frequent right‐sided CDH in patients with left‐sided obstructive cardiac defects such as aortic arch obstruction. In a case–control study comparing prevalence of congenital heart diseases in patients with CDH, there was a trend toward more frequent left‐sided obstructive defects with aortic arch obstruction being 75 times more prevalent than the general population 26. Although the underlying cause for these trends is still unclear, they raise the possibility of a genetic association.

Finally, in our case, hydrops fetalis was also a feature in that the infant demonstrated bilateral pleural effusion and ascites, requiring catheter drainage. As alluded to in the introduction, hydrops fetalis as reported in a 10‐year case series is associated with reduced survival. Other features of note included right‐sided CDH lesions, liver herniation, and lethal anomalies 3. Two cases of non‐immune hydrops were reported by Zankl and colleagues in association with right‐sided diaphragmatic eventration (which involves displacement of the weakened diaphragm with liver and bowel into the thoracic cavity with intact membranous covering) 27. The authors postulated that liver dysfunction resulting from impaired venous return, congestion could have aggravated hypoproteinemia leading to the occurrence of tissue edema. This could have been the mechanism in our case.

With the advent of microarray, deletions and duplications have been increasingly identified as the cause of major congenital malformations. We have reported a case of CDH in a female infant with confirmed interstitial deletion of chromosome 1 with dysmorphic features consistent with previous cases. This is the third case associated with 1p microdeletions raising the possibility of a recurrent genetic predisposition to CDH.

## Conflict of Interest

All authors declare no conflict of interest.

## Authorship

MI: wrote the first draft of this manuscript and was involved in the revisions. She was in the clinical team caring for the infant. MH: provided clinical genetics expertise and assisted with the first draft of manuscript and its subsequent revisions. LG: performed the genetic laboratory testing and provided input about the microarray analysis. YC: performed the autopsy and provided information for the postmortem findings. AM: provided medical care and provided input about clinical details for the manuscript. AS: provided medical care and provided input about clinical details for the manuscript. KT: provided medical care and assisted with the first draft of manuscript and subsequent revisions.

## Supporting information


**Table S1.** OMIM genes identified within the deleted interval where no associated OMIM phenotype map key information was available.
