# Supplementary material for: Interstitial deletion of chromosome 1 (1p21.1p12) in an infant with congenital diaphragmatic hernia, hydrops fetalis, and interrupted aortic arch
Source: Clin Case Rep. 2017 Jan 23;5(2):164–9. doi: 10.1002/ccr3.759 (PMC5290521; doi:10.1002/ccr3.759)
Supplement: Supplementary file 1 — Table S1. OMIM genes identified within the deleted interval where no associated OMIM phenotype map key information was available. [file CCR3-5-164-s001.docx]

**SUPPLEMENTARY TABLES**

**Table 1.** OMIM genes identified within the deleted interval where no associated OMIM phenotype map key information was available.

| **No.** | **OMIM** | **Gene symbols** |
| --- | --- | --- |
| 1 | [608274](http://www.omim.org/entry/608274) Protein arginine N-methyltransferase 6 | PRMT6 |
| 2 | [613994](http://www.omim.org/entry/613994) Neuroblastoma breakpoint family, member 4 | NBPF4 |
| 3 | [176844](http://www.omim.org/entry/176844) Proteasome component 5 | PSMA5, PSC5 |
| 4 | [176262](http://www.omim.org/entry/176262) | - |
| 5 | [606168](http://www.omim.org/entry/606168) DEAD/H box 20 | DDX20 |
| 6 | [607718](http://www.omim.org/entry/607718) Synaptotagmin 6 | SYT6 |
| 7 | [153420](http://www.omim.org/entry/153420) CD58 antigen (lymphocyte function-associated antigen 3) | CD58, LFA3 |
| 8 | [104660](http://www.omim.org/entry/104660) Amylase, pancreatic, alpha-2B | AMY2B |
| 9 | [608818](http://www.omim.org/entry/608818) Netrin G1 | NTNG1, LMNT1, KIAA0976 |
| 10 | [612178](http://www.omim.org/entry/612178) Hen1 methyltransferase, arabidopsis, homolog of | HENMT1, C1orf59, HEN1 |
| 11 | [120420](http://www.omim.org/entry/120420) Colony-stimulating factor-1 (macrophage) | CSF1, MCSF |
| 12 | [613360](http://www.omim.org/entry/613360) Damage-regulated autophagy modulator 2 | DRAM2, TMEM77 |
| 13 | [615100](http://www.omim.org/entry/615100) CTTNBP2 N terminus-like protein | CTTNBP2NL |
| 14 | [604950](http://www.omim.org/entry/604950) Putative homeodomain transcription factor 1 | PHTF1 |
| 15 | [602162](http://www.omim.org/entry/602162) Synaptonemal complex protein-1 | SYCP1, SCP1 |
| 16 | [182310](http://www.omim.org/entry/182310) ATPase, Na+K+ transporting, alpha-1 polypeptide | ATP1A1 |
| 17 | [613952](http://www.omim.org/entry/613952) Family with sequence similarity 46, member C | FAM46C |
| 18 | [104650](http://www.omim.org/entry/104650) Amylase, pancreatic, alpha-2A | AMY2A |
| 19 | [605541](http://www.omim.org/entry/605541) Vav3 oncogene | VAV3 |
| 20 | [600774](http://www.omim.org/entry/600774) | - |
| 21 | [176265](http://www.omim.org/entry/176265) Potassium voltage-gated channel, Shaw-related subfamily, member 4 | KCNC4 |
| 22 | [614234](http://www.omim.org/entry/614234) Chromosome 1 open reading frame 88 (pitchfork, mouse, homolog of) | C1orf88, PIFO |
| 23 | [601968](http://www.omim.org/entry/601968) Wingless-type MMTV integration site family, member 13 | WNT13, XWNT2 |
| 24 | [615858](http://www.omim.org/entry/615858) Round spermatid basic protein 1 | RSBN1, ROSBIN |
| 25 | [603491](http://www.omim.org/entry/603491) Immunoglobulin superfamily, member 3 | IGSF3 |
| 26 | [104700](http://www.omim.org/entry/104700) Amylase, salivary, alpha-1A | AMY1A |
| 27 | [608744](http://www.omim.org/entry/608744) | - |
| 28 | [602420](http://www.omim.org/entry/602420) Potassium voltage-gated channel, shaker-related subfamily, member 10 | KCNA10 |
| 29 | [179520](http://www.omim.org/entry/179520) RAS-related protein RAP1A | RAP1A, KREV1 |
| 30 | [601204](http://www.omim.org/entry/601204) Prostaglandin F2 receptor negative regulator | PTGFRN, FPRP |
| 31 | [104702](http://www.omim.org/entry/104702) Amylase, salivary, alpha-1C | AMY1C |
| 32 | [613996](http://www.omim.org/entry/613996) Neuroblastoma breakpoint family, member 6 | NBPF6 |
| 33 | [611189](http://www.omim.org/entry/611189) Micro rNA 197 | MIR197 |
| 34 | [151525](http://www.omim.org/entry/151525) CD53 antigen | CD53, MOX44 |
| 35 | [601580](http://www.omim.org/entry/601580) Capping protein, muscle Z-line, alpha-1 | CAPZA1, CAPPA1 |
| 36 | [613133](http://www.omim.org/entry/613133) Tetraspanin 2 | TSPAN2, NET3 |
| 37 | [186990](http://www.omim.org/entry/186990) CD2 antigen (p50), sheep red blood cell receptor | CD2 |
| 38 | [104701](http://www.omim.org/entry/104701) Amylase, salivary, alpha-1B | AMY1B |
| 39 | [608339](http://www.omim.org/entry/608339) | - |
| 40 | [607826](http://www.omim.org/entry/607826) | - |
| 41 | [615111](http://www.omim.org/entry/615111) DENN/MADD domain-containing protein 2D | DENND2D |
| 42 | [610742](http://www.omim.org/entry/610742) Moloney leukemia virus 10, mouse, homolog of | MOV10, KIAA1631 |
| 43 | [604516](http://www.omim.org/entry/604516) | - |
| 44 | [601526](http://www.omim.org/entry/601526) Chitinase 3-like 2 | CHI3L2, YKL39 |
| 45 | [165380](http://www.omim.org/entry/165380) RAS homolog gene family, member C (oncogene RHO H9) | RHOC, ARHC, ARH9, RHOH9 |
| 46 | [609683](http://www.omim.org/entry/609683) DNA cross-link repair protein 1B | DCLRE1B, SNM1B, APOLLO |
| 47 | [162361](http://www.omim.org/entry/162361) Nescient helix loop helix 2 | NHLH2, HEN2 |
| 48 | [604718](http://www.omim.org/entry/604718) Transcription termination factor, RNA polymerase II | TTF2 |
| 49 | [615734](http://www.omim.org/entry/615734) WD repeat-containing protein 47 | WDR47, NEMITIN, KIAA0893 |
| 50 | [610299](http://www.omim.org/entry/610299) Solute carrier family 6 (neurotransmitter transporter), member 17 | SLC6A17, NTT4 |
| 51 | [606080](http://www.omim.org/entry/606080) Chitinase, acidic | CHIA, TSA1902, CHIT2 |
| 52 | [609957](http://www.omim.org/entry/609957) Protein phosphatase, magnesium-dependent, 1J | PPM1J, PP2CZ, PPP2CZ |
| 53 | [608003](http://www.omim.org/entry/608003) | - |
| 54 | [608275](http://www.omim.org/entry/608275) Solute carrier family 22 (organic cation transporter), member 15 | SLC22A15, FLIPT1 |
| 55 | [604345](http://www.omim.org/entry/604345) Mannosidase, alpha, class 1A, member 2 | MAN1A2 |
| 56 | [611298](http://www.omim.org/entry/611298) KIAA1324 gene | KIAA1324, EIG121 |
| 57 | [603270](http://www.omim.org/entry/603270) | - |
| 58 | [615943](http://www.omim.org/entry/615943) | - |
| 59 | [609318](http://www.omim.org/entry/609318) Tripartite motif-containing protein 45 | TRIM45 |
| 60 | [607529](http://www.omim.org/entry/607529) Seryl-tRNA synthetase | SARS, SERS |
| 61 | [603878](http://www.omim.org/entry/603878) | - |
| 62 | [610088](http://www.omim.org/entry/610088) Olfactomedin-like 3 | OLFML3, OLF44 |
| 63 | [608162](http://www.omim.org/entry/608162) V-set domain containing T cell activation inhibitor 1 | VCTN1, B7H4, B7X, B7S1 |
| 64 | [604265](http://www.omim.org/entry/604265) Cadherin EGF LAG seven-pass G-type receptor 3 (epidermal growth factor-like 2) | CELSR2, EGFL2, MEGF3 |
| 65 | [608521](http://www.omim.org/entry/608521) Hepatitis B virus X protein-interacting protein | HBXIP |
| 66 | [605783](http://www.omim.org/entry/605783) Breast carcinoma amplified sequence 2 | BCAS2, DAM1 |
| 67 | [613126](http://www.omim.org/entry/613126) Proline/serine-rich coiled-coil protein 1 | PSRC1, DDA3 |
| 68 | [606233](http://www.omim.org/entry/606233) Prokineticin 1 | PROK1, PK1, PRK1, EGVEGF |
| 69 | [615689](http://www.omim.org/entry/615689) Adhesion molecule with Ig-like domain 1 | AMIGO1, ALI2, KIAA1163 |
| 70 | [176263](http://www.omim.org/entry/176263) Potassium voltage-gated channel, shaker-related subfamily, member 3 | KCNA3 |
| 71 | [606916](http://www.omim.org/entry/606916) G protein-coupled receptor 61 | GPR61 |
| 72 | [615354](http://www.omim.org/entry/615354) Ligand-dependent nuclear receptor-interacting factor 1 | LRIF1, RIF1 |
| 73 | [191510](http://www.omim.org/entry/191510) Cold-shock domain-containing E1, RNA-binding | CSDE1, D1S155E, UNR |
| 74 | [603578](http://www.omim.org/entry/603578) Oviductal glycoprotein 1 | OVGP1 |
| 75 | [611656](http://www.omim.org/entry/611656) Suppressor of IKK-epsilon | SIKE |
| 76 | [611734](http://www.omim.org/entry/611734) WD repeat-containing protein 77 | WDR77, MEP50 |
| 77 | [600445](http://www.omim.org/entry/600445) Adenosine A3 receptor | ADORA3 |
| 78 | [138333](http://www.omim.org/entry/138333) Glutathione S-transferase M4 | GSTM4 |
| 79 | [138380](http://www.omim.org/entry/138380) Glutathione S-transferase M2, muscle | GSTM2, GST4 |
| 80 | [138350](http://www.omim.org/entry/138350) Glutathione S-transferase M1 | GSTM1 |
| 81 | [138385](http://www.omim.org/entry/138385) Glutathione S-transferase M5 | GSTM5 |
| 82 | [138390](http://www.omim.org/entry/138390) Glutathione S-transferase M3, brain | GSTM3, GST5 |
| 83 | [614989](http://www.omim.org/entry/614989) EPS8-like protein 3 | EPS8L3, EPS8R3 |
| 84 | [611127](http://www.omim.org/entry/611127) Ubiquitin-like 4B | UBL4B |
